# Prevalence of childhood and early adolescence mental disorders among children attending primary health care centers in Mosul, Iraq: a cross-sectional study

**DOI:** 10.1186/1471-2458-7-274

**Published:** 2007-10-02

**Authors:** Asma A Al-Jawadi, Shatha Abdul-Rhman

**Affiliations:** 1 Department of Community Medicine, College of Medicine, University of Mosul, Mosul, Iraq; 2 College of Nursing, University of Mosul, Mosul, Iraq

## Abstract

**Background:**

Children and adolescents are more vulnerable to the affects of war and violence than adults. At the time of initiation of this study, nothing was known about the prevalence of childhood and early adolescence mental disorders. The aim of the present study is to measure the point prevalence of mental disorders among children of 1–15 years age in the city of Mosul, Iraq.

**Methods:**

A cross-sectional study design was adopted. Four primary health care centers were chosen consecutively as a study setting. The subjects of the present study were mothers who came to the primary health care center for vaccination of their children. The chosen mothers were included by systematic sampling randomization. All children (aged 1–15) that each mother had were considered in the interview and examination.

**Results:**

Out of 3079 children assessed, 1152 have childhood mental disorders, giving a point prevalence of 37.4%, with a male to female ratio of to 1.22:1. The top 10 disorders among the examined children are post-traumatic stress disorder (10.5%), enuresis (6%), separation anxiety disorder (4.3%), specific phobia (3.3%) stuttering and refusal to attend school (3.2% each), learning and conduct disorders (2.5% each), stereotypic movement (2.3%) and feeding disorder in infancy or early childhood (2.0%). Overall, the highest prevalence of mental disorders was among children 10–15 years old (49.2%) while the lowest was among 1–5 year olds (29.1%). Boys are more affected than girls (40.2% and 33.2%, respectively).

**Conclusion:**

Childhood mental disorders are a common condition highly prevalent amongst the children and early adolescents in Mosul. Data from the present study mirrors the size of the problem in local community. Several points deserve attention, the most important of which include giving care at the community level, educating the public on mental health, involving communities and families, monitoring community mental health indicators, and providing treatment at primary health care level.

## Background

In 1946 the World Health Organization (WHO) defined health as a state of complete physical, mental and social wellbeing, and not merely the absence of disease or infirmity. This definition provides a base for the broad perspective needed to analyze a population health [1].

Mental disorders are common, affecting more than 25% of all people at some time during their lives. They are also universal, affecting people of all countries and societies. They have an economic impact on society and on the quality of life of individuals and families. Mental disorders are present at any point in time in about 10% of the adult population. It was estimated that, in 1990, mental and neurological disorders accounted for 10% of the total disability-adjusted life years (DALYs) lost to all diseases and injuries. By 2000 this had risen to 12%, and by 2020 it is projected that the burden of these disorders will increase to 15% [2]. Common disorders that usually cause severe disabilities are: depression, substance abuse, schizophrenia, epilepsy, mental retardation (MR) and disorders of childhood and adolescence [3]. The overall prevalence of mental and behavioral disorders among children has been investigated in several studies from developed and developing countries. Though the prevalence figures vary considerably among studies, it appears that 10–20% of all children have one or more mental or behavioral problems [2].

Mental and behavioral disorders of childhood and adolescence are very costly in both human and financial terms. The aggregate disease burden of these disorders has not been estimated, and it would be complex to calculate because many of these disorders can be precursors to much more disabling disorders during later life [2].

For more than two decades, the Iraqi nation as a whole has been suffering from wars, sanctions and urban violence. The Iraqi children have been so greatly affected by these dire conditions that we suspect they are facing very real dangers of disease, starvation, psychological trauma and death. A report released by a Canadian-led team of health experts stated that the children in Iraq have a great fear of war. Children as young as four described their clear opinion about the horrors of war. They are afraid, anxious, and depressed about the prospect of war, the report says. Many have nightmares, and only 40% think that life is worth living [4]. These statements were documented in 2003. Before that time, other studies were carried out that were mainly concerned with the effect of trauma on refugee children or in the post conflict areas in Kurdistan from 2000 onwards [5-7]. Many other studies investigated mental disorders among adult refugees in USA and Netherlands [8,9].

At the time of initiation of this study, there were no available data to describe the situation of mental disorders among children aged 1–15 years old in Mosul, one of the largest cities in Iraq after the capital, Baghdad. Mosul is a highly populated city, where 39.8% of its population are children aged 1–15 years old. In addition, it has a high annual rate of growth that is similar to the overall rate of growth in Iraq (2.9%). This warrants an active approach to establish the overall point prevalence of mental disorders among children and adolescents aged 1–15 years old, and to determine the rank order of each specific disorder.

## Methods

### Ethical agreement

Prior to data collection, official written permission was obtained from the ethical and scientific committee of the Directorate of Health (DOH) of Ninevah. Prior informed consent from the mothers of all children in the studywas orally obtained after an explanation of the aims and objectives of the work.

### Study setting

The study was conducted in Mosul city, the center of the Ninevah governorate in the north of Iraq. For the purpose of data collection, and because of poor security conditions meaning it was impossible to carry out a household survey, a multistage sampling method was used. As Mosul is situated on both sides of the River Tigris, two primary health care centers (PHCCs) were consecutively chosen from the official health sectors on each side. The chosen centers have wide catchments areas and they serve a population whose size varies between 32676–60390 with differing socio-economic stratas.

### Study participants

According to the national strategy of the Iraqi Ministry of Health, all children present in the catchment areas of PHCCs should receive vaccination at those centers. All the mothers (n = 843) included in the study by systematic sampling randomization (where every third woman was accepted) were informed about the study and requested by the interviewer to bring their children aged between 1–15 (if they had any) to the center on a fixed date.

**Figure 1:**
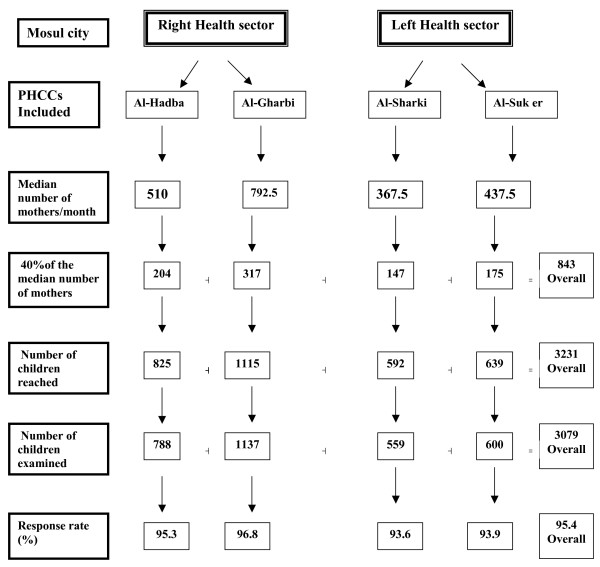
Sample size determination in the present study.

Figure 1 illustrates the sample size determination in the present study. The median number of mothers attending each primary health care center (PHCC) per month was considered as a background. Then, 40% of this number was taken, which yielded 3231 children aged between 1–15 years old. Non-participants included refusals to participate, no-shows and non-eligible children. Thus, the final sample included 3079 children. No information was collected about the non-participants.

### Data collection

Data collection was performed by one interviewer using a standardized questionnaire form. The items on this form included the diagnostic criteria taken from DSM-IV-TR2000 [10]. Refusal to attend school was diagnosed according to the criteria mentioned in DSM-III-1980 [11]. The intent of the investigators was to classify refusal to attend school into intentional and obligatory. The designed form was reviewed by a scientific committee consisting of six experts, two from the fields of psychiatry, three from community medicine and one from health statistics. During the pilot study, this questionnaire was shown to have a high reliability (83.5%) and validity (89.6%).

The respondent mother and her child or children were interviewed in a special room in the PHCC. The interviewer inquired about the socio-demographic characteristics of the family, the child's developmental history, past medical history, history of mental disorders among the family, the child's toys, interest and hobbies. In addition, a child interview was performed with children greater than 7 years old. With children aged 1–7 years old, a simple interview was conducted according to the age. Any child who had any impairment or delay in development of specific functions such as speech or language, or overall pervasive development, or any disturbance in behavior or emotion was considered as a case of mental disorder and hence included in the mental disorder group.

Data collection was conducted over the eight-month period from 1 September 2003 to 30 April 2004. Data entry and statistical analysis was carried out using the SPSS package. The prevalence objective of this study was analyzed as the proportion of children who have mental disorders out of the total surveyed population, by age group and sex.

Chi-square and Z-tests were used to find statistical association or difference. For the presence or absence of significance, a p-value < 0.05 was considered to be significant throughout the study analysis.

## Results

During the eight-month period of data collection, 843 mothers were interviewed. This figure yielded a total of 3231 children aged 1–15 years old. Of this number, 3079 children were included in the study.

Table 1 shows the socio-demographic characteristics of study population. Over a quarter (26.9%) of the children were 1–4 years old, 44.4% were 5–9 years old and 28.7% were 10–15 years old. The male to female ratio was 1.22:1. Housewives formed 95.8% of the total mothers approached; 92.3% of fathers were employed (either self-employed, civil servants or in the private sector). A total of 67.4% of mothers were either illiterate or had only a primary certificate of education, whereas 23.2% of fathers were university graduates.

**Table 1:** Socio-demographic characteristics of the study sample

**Characteristic**		**Total no. of children**	**%**
Age	1–4	829	26.9
	5–9	1367	44.4
	10–15	883	28.7
	Total	3097	100.0
Gender	Male	1698	55.1
	Female	1381	44.9
	Total	3097	100.0
Occupation of mother	Housewife	2951	95.8
	Working	128	4.2
	Total	3097	100.0
Occupation of father	Working	2841	92.3
	Not working	238	7.7
	Total	3097	100.0
Education of mother	Illiterate	771	25.0
	Primary	1305	42.4
	Intermediate	552	17.9
	Secondary	152	4.9
	University	294	9.5
	Postgraduate	5	0.2
	Total	3079	100.0
Education of father	Illiterate	427	13.9
	Primary	991	32.2
	Intermediate	612	19.9
	Secondary	334	10.8
	University	653	21.2
	Postgraduate	62	2.0
	Total	3079	100.0

At the time of the survey there were 1152 diseased children in the study population. The overall point prevalence of mental disorders among the study sample was 37.4%. The top 10 disorders found were: post-traumatic stress disorder (PTSD; 10.5%), enuresis (6.0%), separation anxiety disorder (4.3%) and specific phobia (3.3%). Stuttering and refusal to attend school each had a point prevalence of 3.2%. The point prevalence of learning disorders (LD) was 2.5%, and the same figure was reported for conduct disorder (CD). Stereotypic movement had a prevalence of (2.3%), and feeding disorder of infancy or early childhood (2.0%).

Gender and type specific point prevalence stratification is shown in Table 2. Overall, mental disorders were significantly more common among boys than girls (40.9% and 33.2%, respectively; p < 0.001). However, PTSD showed a reverse trend, where girls had a significantly higher point prevalence than boys (13.8% versus 7.8%; p < 0.001). Specific phobia showed the same trend (4.9% versus 2.1%) as did rumination (0.7% versus 0.4%). Enuresis is significantly more common among boys (p = 0.008). Similarly, stuttering (p < 0.001), conduct disorders (p < 0.001) and feeding disorders of infancy or early childhood (p = 0.011) were more common in boys.

**Table 2:** Gender specific point prevalence (%) of childhood and early adolescence mental disorders among the study sample

	**Boys**		**Girls**		**Total**	
	
**Disorders**	No.	%	No.	%	No.	%
PTSD	132	7.8	190	13.8	322	10.5
Enuresis	120	7.1	66	4.8	186	6.0
Separation anxiety disorder	71	4.2	62	4.5	133	4.3
Specific phobia	35	2.1	68	4.9	103	3.3
Stuttering	74	4.4	25	1.8	99	3.2
Refusal to attend school	60	3.5	38	2.8	98	3.2
Learning disorder	45	2.7	32	2.3	77	2.5
Conduct disorder	62	3.7	14	1.0	76	2.5
Stereotypic movement	40	2.4	32	2.3	72	2.3
Feeding disorder of infancy or early childhood	44	2.6	18	1.3	62	2.0
Mental retardation	35	2.1	23	1.7	58	1.9
Pica	33	1.9	23	1.7	56	1.8
Reactive attachment disorder of infancy or early childhood	32	1.9	22	1.6	54	1.8
Communication	32	1.9	19	1.4	51	1.7
Oppositional defiance disorder	42	2.5	11	0.8	53	1.7
Phonological disorder	35	2.1	15	1.1	50	1.6
Depression	21	1.2	26	1.9	47	1.5
ADHD	35	2.1	4	0.3	39	1.3
Tic disorder	25	1.5	12	0.9	37	1.2
Rumination	7	0.4	10	0.7	17	0.6
Motor skill disorder	2	0.12	1	0.07	3	0.1
Autism	2	0.12	1	0.07	3	0.1
Selective mutism	2	0.1	2	0.1	4	0.1
Encopresis	1	0.1	0	0.0	1	0.03

Total disordered children	694	40.9	458	33.2	1152	37.4

Total children examined	1698	55.1	1381	44.9	3079	100.0

Table 3 depicts the age specific point prevalence of childhood and early adolescence mental disorders among the study sample. Overall, the highest prevalence was among children 10–15 year old (49.2%), followed by those 5–9 years old (34.9%). The lowest figure was among those 1–4 years old (29.1%). This variation is of highly significant value (p < 0.001). Considering the older age group, separation anxiety disorder and PTSD were at the top of the list (11.2% and 11.0%) respectively, while for the age range 5–9, PTSD and enuresis had the highest values (9.7% and 8.9%, respectively). PTSD remains the disease of highest prevalence in the 1–4 year old age group (11.1%), followed by reactive attachment disorder of infancy or early childhood (6.5%).

**Table 3:** Age specific point prevalence (%) of childhood and early adolescence mental disorders among the study sample

	**Age (years)**							
	
	**1–4**		**5–9**		**10–15**		**Total**	
	
**Disorder**	**No.**	**%**	**No.**	**%**	**No.**	**%**	**No.**	**%**
PTSD	92	11.1	133	9.7	97	11.0	322	10.5
Enuresis	0	0.0	121	8.9	65	7.4	186	6.0
Separation anxiety disorder	7	0.8	27	2.0	99	11.2	133	4.3
Specific phobia	0	0.0	68	5.0	35	4.0	103	3.3
Stuttering	32	3.9	47	3.4	20	2.3	99	3.2
Refusal to attend school	0	0.0	34	2.5	64	7.2	98	3.2
Learning disorder	1	0.1	31	2.3	45	5.1	77	2.5
Conduct disorder	7	0.8	33	2.4	36	4.1	76	2.5
Stereotypic movement	45	5.4	20	1.5	7	0.8	72	2.3
Feeding disorder of infancy or early childhood	41	4.9	17	1.2	4	0.5	62	2.0
Mental retardation	18	2.2	18	1.3	22	2.5	58	1.9
Pica	35	4.2	17	1.2	4	0.5	56	1.8
Reactive attachment disorder of infancy or early childhood	54	6.5	0	0.0	0	0.0	54	1.8
Communication disorders	27	3.3	18	1.3	6	0.7	51	1.7
Oppositional defiance disorder	3	0.4	20	1.5	30	3.4	53	1.7
Phonological disorder	25	3.0	13	1.0	12	1.4	50	1.6
Depression	4	0.5	13	1.0	30	3.4	47	1.5
ADHD	0	0.0	17	1.2	22	2.5	39	1.3
Tic disorder	10	1.2	18	1.3	9	1.0	37	1.2
Rumination	11	1.3	4	0.3	2	0.2	17	0.6
Motor skills disorder	1	0.1	2	0.1	0	0.0	3	0.1
Autism	1	0.1	1	0.1	1	0.1	3	0.1
Selective mutism	1	0.1	1	0.1	2	0.2	4	0.1
Encopresis	0	0.0	1	0.1	0	0.0	1	0.03

Total disordered children	241	29.1	477	34.9	434	49.2	1152	37.4

Total children examined	829	26.9	1367	44.4	883	28.7	3079	100.0

Table 4 demonstrates the patterns of comorbidity among the study sample. Out of the total number of children examined, 62.1% have a solitary disorder, 29.9% have two disorders and only 8.0% have more than two. In reference to PTSD, which was the commonest, we found out that among the 127 cases that had two disorders, 24.4% were associated with enuresis, 11.0% with stuttering, 10.2% with refusal to attend school and 9.4% with depression. Other comorbidities showed inconspicuous trends.

**Table 4:** Co-morbidity pattern of childhood and early adolescence mental disorders among the study sample

	**Co-morbidity**							
	
	**One disorder**		**Two disorders**		**More than two disorders**		**Total**	
	
**Disorder**	**No.**	**%**	**No.**	**%**	**No.**	**%**	**No.**	**%**
PTSD	168	52.2	127	39.4	27	8.4	322	100
Enuresis	72	38.7	78	41.9	36	19.4	186	100
Separation anxiety disorder	67	50.4	52	39.1	14	10.5	133	100
Specific phobia	43	41.7	40	38.8	20	19.4	103	100
Stuttering	21	21.2	63	63.6	15	15.2	99	100
Refusal to attend school	39	39.8	37	37.8	22	22.4	98	100
Learning disorder	29	37.7	22	28.6	26	33.8	77	100
Conduct disorder	33	43.4	30	39.5	13	17.1	76	100
Stereotypic movement	47	65.3	21	29.2	4	5.6	72	100
Feeding disorder of infancy or early childhood	28	45.2	15	24.2	19	30.6	62	100
Mental retardation	35	60.3	23	39.7	0	0.0	58	100
Pica	25	44.6	22	39.3	9	16.1	56	100
Reactive attachment disorder of infancy or early childhood	23	42.6	21	38.9	10	18.5	54	100
Communication disorder	15	29.4	23	45.1	13	25.5	51	100
Oppositional defiance disorder	18	34.0	23	43.4	12	22.6	53	100
Phonological disorder	11	22.0	22	44.0	17	34.0	50	100
Depression	14	29.8	24	51.1	11	23.4	47	100
ADHD	12	30.8	16	41.0	11	28.2	39	100
Tic disorder	6	16.2	20	54.1	11	29.7	37	100
Rumination	6	35.3	9	52.9	2	11.8	17	100
Others	3	27.3	4	36.4	4	36.4	11	100

Total diseased	**715**	**62.1**	**345**	**29.9**	**92**	**8.0**	**1152**	**100**

## Discussion

Contrary to popular belief, mental disorders are common during childhood and adolescence. Inadequate attention is paid to this area of mental health [2]. In a recent report, the Surgeon General of the United States has said that the United States is facing a public crisis in the mental health of infants, children and adolescents [12]. According to this report, 1 in 10 young people suffers from mental illness severe enough to cause some level of impairment, yet fewer than 1 in 5 receives the necessary treatment [12]. The situation in large parts of the developing world, including Iraq, is likely to be even worse.

For more than two decades, Iraq has faced many wars and sanctions that have affected all aspects of life, particularly the health status of children and women of childbearing age who are at grave risk of malnutrition, disease, death and mental disorders. Therefore, the point prevalence of childhood and early adolescence mental disorders reported in this study (37.4%) could be an important indicator of the impact of the problem on children in Mosul. If this figure is applied to all children in the population aged 1–15 in Mosul city (n = 180465), this means that at the time of the survey there were 67674 cases of mental disorders amongst this group. In 2007, this figure will probably be much higher. A higher figure was reported in a study carried out on the epidemiology of behavioral and emotional problems among Palestinian children. This study enrolled 959 children from Gaza strip. The results showed that the case incidence was 54.4% in boys and 46.5% girls [13]. The same gender difference has been found in the present study, where the overall prevalence is 40.9% in boys and 33.2% in girls (p < 0.001). Child mental disorder diagnoses vary all over the world; therefore, any diagnosis should take into consideration the culture and the environment where a child is raised, because what is considered normal in one society is considered abnormal in the other. Mental health of children and adolescents is influenced by displacement through wars, catastrophe, by stresses on the family and by economic adversities. The lives of many children who face uncertain futures, including those who are traumatized by wars and disasters, are afflicted by the burden of serious emotional and behavioral disorders [2].

Post-traumatic stress disorder arises after stressful events of an exceptionally threatening or catastrophic nature. The point prevalence of PTSD in the general population, according to the Global Burden of Disease for the year 2000 (GBD2000), is 0.37% [2]. Although the events that cause PTSD are common, there are no epidemiological studies that specifically study PTSD in young persons. It is quite clear, however, that this disorder is more common in young people exposed to life-threatening events [14]. In this study, PTSD is at the top of the list of mental disorders affecting the study sample, where just over 1 in every 10 youths (10.5%) have PTSD. In January to March 2006, three studies on the prevalence of mental disorders among children were performed in Baghdad, Mosul and Dohuk. In Baghdad, 14% of the examined children had PTSD. In Mosul, a very much higher figure was found where 30% of studied subjects had symptoms of PTSD [15]. Previously, a 26 month follow up of PTSD in children after a mass escape in Kurdistan in the north of Iraq showed that 20% of the children aged 5–16 fulfilled the criteria of PTSD at the baseline test. Twenty-six months later 18% were re-identified [5]. Regarding the implications of elevated PTSD for later adult outcomes of afflicted youth, Garbarino and Kostelny [16] in their study of Palestinian mothers and children found that children exposed to the full range of war-related risks generally were well adjusted if they faced these risks in the context of a functional and supportive family system; this is very common among Iraqi families. However, if these children experienced family problems as well as war risk, they would likely suffer more serious psychological distress [16].

The specific diagnosis of PTSD has been questioned as being culture-specific and also being overdiagnosed. Indeed, PTSD has been called a diagnostic category that has been invested based on socio-political needs [17]. However, a comparative study between refugee children in Sweden and Swedish children of the same age, sex, and trauma score showed no significant difference in the PTSD diagnosis [6]. In PTSD, it has been reported that girls always have higher symptom scores than boys. This statement supports the finding of the present study, where girls reported a significantly higher prevalence than boys (13.8% versus 7.8%; p < 0.001). Assessment of 1090 adolescents carried out in Mosul in 2006 showed the same gender difference; however, the reported figures were higher (32% for girls versus 26% for boys) [15]. Age range difference is likely to be responsible for this variation between the results, or it is possible the mothers have significantly underestimated both the intensity and duration of the stress reaction in the present study sample.

The second most common mental disorder found among study sample was enuresis, which had an overall point prevalence of 6.0%. Boys reported significantly higher figures than girls (7.1% versus 4.8%) (p < 0.001). The highest prevalence was among 5–9 year olds (8.9%), then 10–15 year olds (7.4%), with a difference of highly significant value (p < 0.001). Mikkelsen [18] cited lower figures, which were 6.7% for boys 7 years of age who were bedwetting at least once a week or more, compared to only 0.5% for girls. Unfortunately, in this study the investigators did not enquire about the frequency of bedwetting.

The present study shows that separation anxiety ranks third among the list of disorders afflicting the examined children, with a point prevalence of 4.3%. Girls reported a slightly higher figure than boys (4.5% and 4.2%, respectively). The point prevalence of this disorder significantly rises from 0.8% in 1–4 year old children to 2.0% in 5–9 year olds and 11.2% in 10–15 year olds (p < 0.001). Costello et al [19] examined the prevalence of separation anxiety disorder in children aged 9–13 years old in the Great Smoky Mountains study (5.7 ± 1%). Subsequently, Sylvester [20] suggested that children with this disorder are more likely to come from low socio-economic backgrounds and single parent homes. The same suggestion can be applied to the present study, where there is a combination of low socio-economic status with the burden of sanctions, war and daily violence that afflict the majority of Iraqis, particularly children. Such an unusual situation may also lead to an increase in the prevalence of specific phobia (3.3%). An evidently lower figure was reported among American children aged 8, 12 and 17 years old; only 1% of them met the criteria for this disorder [21]. In the present study, the gender distribution of specific phobia is significantly in favor of girls (p < 0.001), and it has a significantly higher rate in the aged 5–9 group (5.0%), improving when the child becomes older (4.0%) (p < 0.001).

Refusal to attend school shows a significantly high prevalence among 10–15 year old children (7.2%; p < 0.001), and a higher rate is reported for boys than girls (3.5% and 2.8%, respectively). Although this gender difference is of no significant value, it is worth noting that this trend is the result of poverty among families where many children at the age of 10–15 tend to leave school. Boys help their fathers to increase the family income, while girls keep house and nurse babies in the absence of their working mothers.

Other less common disorders show either an increase among children aged 1–4 years old, or start to appear at this age and become very evident among 10–15 year olds. For all these disorders, boys are more affected than girls except for depression and rumination disorders.

### Methodology issues

There are certain points relevant to this study that merit discussion. This study is cross-sectional in design. Because of the poor security conditions in Mosul, the study sample was based at the PHCC that serves as the primary care provider. This may not represent the whole population. Accounting for failure to show, refusals to participate and ineligibility, the estimated participation rate is approximately 95.4%. Unfortunately, no information was collected about non-participants. In the present work, the study unit was mothers who attended the PHCC for vaccination of their children. This was chosen for two reasons: the first was to overcome the bias of sampling based on PHCC, as all the children present in the catchment area of the PHCC are to have their vaccination there and mothers are responsible for bringing them to the PHCC. Therefore, mothers were asked by the interviewer to bring their other children aged 1–15 to the center. The second reason was to ensure ease of interviewing the children aged 1–7 years old at the PHCC, as at this age the interview needs to take place in a location the child is comfortable in. The well-designed questionnaire, which was introduced by a well-trained interviewer in a friendly atmosphere, might have reduced this bias.

In the present study, the specific mental disorders were diagnosed according to DSM-IV-TR 2000 criteria [10]. In Mosul city there are no facilities to diagnose different grades of mental retardation, types of learning disorder and communication disorders; thus, the diagnoses was made as a specific disorder only (i.e., the further specification of these three categories was not carried out).

## Conclusion

The present study concludes that childhood and early adolescence mental disorders are common conditions afflicting children and early adolescents in Mosul. Several points are important for consideration: the giving of care at a community level; public education on mental health, involving families and communities in decision-making on policies, programs and services; and the inclusion of mental health indicators in the health records of the Ministry of Health. Lastly, provision of care and treatment at the primary health care level is urgently needed.

## Competing interests

The author(s) declare that they have no competing interests.

## Authors' contributions

Both authors contributed equally in designing and conducting the survey, analyzing data, and drafting the manuscript. Both authors read and approved the current draft.

## Pre-publication history

The pre-publication history for this paper can be accessed here:


